# A dataset for Wi-Fi-based human-to-human interaction recognition

**DOI:** 10.1016/j.dib.2020.105668

**Published:** 2020-05-11

**Authors:** Rami Alazrai, Ali Awad, Baha’A. Alsaify, Mohammad Hababeh, Mohammad I. Daoud

**Affiliations:** a Department of Computer Engineering, German Jordanian University, P.O. Box 35247, Amman 11180, Jordan; b Department of Network Engineering and Security, Jordan University of Science and Technology, P.O. Box 3030, Irbid 22110, Jordan

**Keywords:** Two-Person Interaction, Wi-Fi, Channel State Information (CSI), Received Signal Strength Indicator (RSSI), Human Activity Recognition

## Abstract

This paper presents a dataset for Wi-Fi-based human-to-human interaction recognition that comprises twelve different interactions performed by 40 different pairs of subjects in an indoor environment. Each pair of subjects performed ten trials of each of the twelve interactions and the total number of trials recorded in our dataset for all the 40 pairs of subjects is 4800 trials (i.e., 40 pairs of subjects × 12 interactions × 10 trials). The publicly available CSI tool [1] is used to record the Wi-Fi signals transmitted from a commercial off-the-shelf access point, namely the Sagemcom 2704 access point, to a desktop computer that is equipped with an Intel 5300 network interface card. The recorded Wi-Fi signals consist of the Received Signal Strength Indicator (RSSI) values and the Channel State Information (CSI) values. Unlike the publicly available Wi-Fi-based human activity datasets, which mainly have focused on activities performed by a single human, our dataset provides a collection of Wi-Fi signals that are recorded for 40 different pairs of subjects while performing twelve two-person interactions. The presented dataset can be exploited to advance Wi-Fi-based human activity recognition in different aspects, such as the use of various machine learning algorithms to recognize different human-to-human interactions.

Specifications table

**Table:** 

Subject	Computer Science Applications Signal Processing Human-Computer Interaction Computer Vision and Pattern Recognition
Specific subject area	Activity recognition, Human-to-human interaction recognition using Wi-Fi signals.
Type of data	Raw dataset, table, image
How data were acquired	The Wi-Fi signals were captured in an indoor environment (furnished room) from 40 different pairs of subjects while performing twelve different human-to-human interactions. The CSI tool [1] was used to record the Wi-Fi signals transmitted from a commercial off-the-shelf access point, namely the Sagemcom 2704 access point, to a desktop computer that is equipped with an Intel 5300 Network Interface Card (NIC). The number of transmit antennas in the utilized access point is two antennas, while the number of receive antennas in the employed NIC is three antennas.
Data format	Raw
Parameters for data collection	The twelve interactions were thoroughly explained to the subjects before the beginning of data recording. All the interactions were performed in a line-of-sight manner with respect to the access point and the NIC. The access point was configured to operate at a frequency band of 2.4 GHz, wireless channel number 6, channel bandwidth of 20 MHz, and a modulation coding scheme of index 8.
Description of data collection	The Wi-Fi signals were recorded from 40 different pairs of subjects while performing twelve different human-to-human interactions. Each pair of subjects performed ten trials of each interaction. The recorded Wi-Fi signals consist of the Received Signal Strength Indicator (RSSI) values and the Channel State Information (CSI) values.
Data source location	Institution: German Jordanian University, Department of Computer Engineering City/Town/Region: Amman, 11180 Country: Jordan Latitude and longitude (and GPS coordinates) for collected samples/data: 31.7767° N, 35.8025° E
Data accessibility	Repository name: Mendeley Data Data identification number: 10.17632/3dhn4xnjxw.1 Direct URL to data: https://data.mendeley.com/datasets/3dhn4xnjxw/draft?a=90c726d4-5493-4efc-9ee6-973bcd922b31

## Value of the data

• The dataset contains a collection of Wi-Fi signals, including the RSSI and CSI values, which are recorded for 40 different pairs of subjects while performing twelve human-to-human interactions in an indoor environment. To the best of our knowledge, this is the first Wi-Fi-based dataset that considers the activities performed by two individuals.
• Researchers in the field of human activity recognition can utilize the acquired data to evaluate the performance of Wi-Fi-based human-to-human interaction recognition systems that can be developed for various application domains.
• The acquired data can be exploited to advance human activity recognition technology in different aspects. For example, various pattern recognition and machine learning methods can be used to accurately recognize different human-to-human interactions. Another potential use of our data is to explore the utilization of various signal processing techniques to analyze the recorded Wi-Fi signals and extract salient features that can be used to recognize different human-to-human interactions.
• The existing publicly available Wi-Fi-based human activity datasets have focused on activities that are performed by a single human. On the contrary, our dataset contributes to the ongoing research in the field of Wi-Fi-based human activity recognition by providing a collection of Wi-Fi signals that are recorded from 40 different pairs of subjects while performing twelve human-to-human interactions.

## 1 Data description

The raw data are grouped into one main folder that comprises 40 subfolders, where each sub-folder contains the data files recorded for a particular pair of subjects. Specifically, a total of 120 trials (i.e., 10 trials per each interaction x 12 interactions) were recorded for each pair of subjects, where each trial was stored in a separate MATLAB data file (.mat).

The name of each data file follows the form “Sx_Sy_In_Tk.mat”. The first part of the name of each data file, denoted as Sx_Sy, represents the pair of subjects who have performed the interaction recorded in a specific data file. In particular, Sx and Sy are the first and second subjects within the pair Sx_Sy, respectively. The pairs of the subjects were formed from a pool of 66 different subjects, as described in the next section. Hence, x and y are integer numbers between 1 and 66. The second part of the name of each data file, denoted as ln, represents one of the twelve human-to-human interactions, where n is an integer number between 1 and 12. Specifically, In = {approaching (I1), departing (I2), handshaking (I3), high five (I4), hugging (I5), kicking with the left leg (I6), kicking with the right leg (I7), pointing with the left hand (I8), pointing with the right hand (I9), punching with the left hand (I10), punching with the right hand (I11), and pushing (I12)}. Finally, the last part of the name of each data file, denoted as Tk, represents the trial number, where k is an integer number between 1 and 10. For example, the data file name “S15_S3_I2_T3.mat” represents a file containing the data recorded for the pair of subjects S15_S3 while performing the departing interaction during the third trial.

The data file associated with each trial contains a cell array of dimension L × 1, where L represents the number of Wi-Fi packets captured during the recording of a particular trial. Moreover, each Wi-Fi packet is stored within an element of the cell array in the form of a structure that consists of several fields as described in Table 1.

**Table 1:** The description of the fields in the stucture that contains a Wi-Fi packet.

Field	Description	
timestamp_low	The arrival time of the Wi-Fi packet, which is represented by the lower 32 bits of NIC's clock [1]. This timestamp also represents the arrival time of the RSSI and CSI values comprised within the Wi-Fi packet.	
Nrx	Nrx represents the number of antennas used at the receiver side (i.e., the NIC) and its value is set to 3.	
Ntx	Ntx represents the number of antennas used at the transmitter side (i.e., the access point) and its value is set to 2.	
noise	The measured noise over the channel.	
agc	Represents the automatic gain control parameter of the NIC measured in dB. The value of this field along with the value in the noise field are necessary to convert the unit of the RSSI values from dB to dBm as described in the CSI tool [1].	
RSSI_a	RSSI_a represents the RSSI value received at the first antenna of the NIC measured in dB.	
RSSI_b	RSSI_b represents the RSSI value received at the second antenna of the NIC measured in dB.	
RSSI_c	RSSI_c represents the RSSI value received at the third antenna of the NIC measured in dB.	
CSI	The channel state information in the form of a complex three-dimensional matrix that has a dimension of Ntx × Nrx × Nsc. Nsc represents the number of subcarriers constructed using the Orthogonal Frequency-Division Multiplexing (OFDM) modulation scheme, which is applied to the utilized 20 MHz wide channel. The CSI tool specifies the value of the Nsc parameter to 30 subcarriers [1].	
label	The recorded trial for any of the twelve human-to-human interactions consists of two types of intervals, namely the steady-state and the interaction intervals. During the steady state interval, the pair of subjects are standing against each other without performing any activity. On the other hand, during the interaction interval, the pair of subjects perform one of the twelve different human-to-human interactions. Thus, this field assigns a label to the Wi-Fi packet to specify whether the packet has arrived during the steady state interval or the interaction interval. In particular, the assigned label is a string of the form In, where n is an integer in the range of 1 to 13 that is assigned to each Wi-Fi packet as follows:	
	**Label**	**Description**
	I1	A Wi-Fi packet is labeled as I1 if it arrived while the pair of subjects were performing the approaching interaction.
	I2	A Wi-Fi packet is labeled as I2 if it arrived while the pair of subjects were performing the departing interaction.
	I3	A Wi-Fi packet is labeled as I3 if it arrived while the pair of subjects were performing the handshaking interaction.
	I4	A Wi-Fi packet is labeled as I4 if it arrived while the pair of subjects were performing the high five interaction.
	I5	A Wi-Fi packet is labeled as I5 if it arrived while the pair of subjects were performing the hugging interaction.
	I6	A Wi-Fi packet is labeled as I6 if it arrived while the pair of subjects were performing the kicking with the left leg interaction.
	I7	A Wi-Fi packet is labeled as I7 if it arrived while the pair of subjects were performing the kicking with the right leg interaction.
	I8	A Wi-Fi packet is labeled as I8 if it arrived while the pair of subjects were performing the pointing with the left hand interaction.
	I9	A Wi-Fi packet is labeled as I9 if it arrived while the pair of subjects were performing the pointing with the right hand interaction.
	I10	A Wi-Fi packet is labeled as I10 if it arrived while the pair of subjects were performing the punching with the left hand interaction.
	I11	A Wi-Fi packet is labeled as I11 if it arrived while the pair of subjects were performing the punching with the right hand interaction.
	I12	A Wi-Fi packet is labeled as I12 if it arrived while the pair of subjects were performing the pushing interaction.
	I13	A Wi-Fi packet is labeled as I13 if it arrived while the pair of subjects were in the steady state interval.

**Fig. 1:**
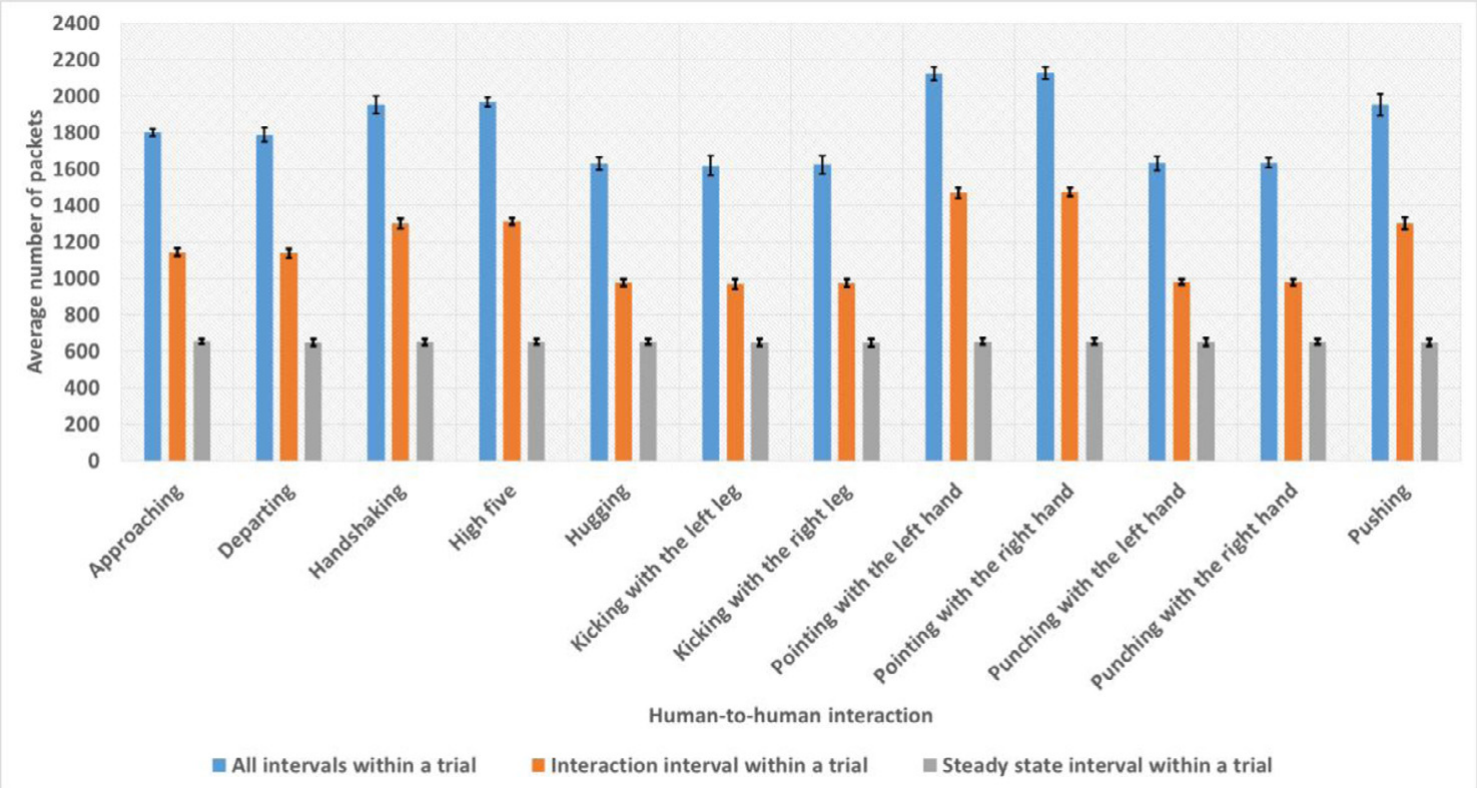
The average number of packets recorded for all the intervals, interaction interval, and steady state interval within a trial computed for each interaction over all the pairs of subjects. The black bars represent the standard deviation values.

Fig. 1 shows the average ± standard deviation number of Wi-Fi packets recorded in all the intervals within a trial, the interaction interval within a trial, and the steady state interval within a trial computed for each of the twelve interactions over all the pairs of subjects.

**Fig. 2:**
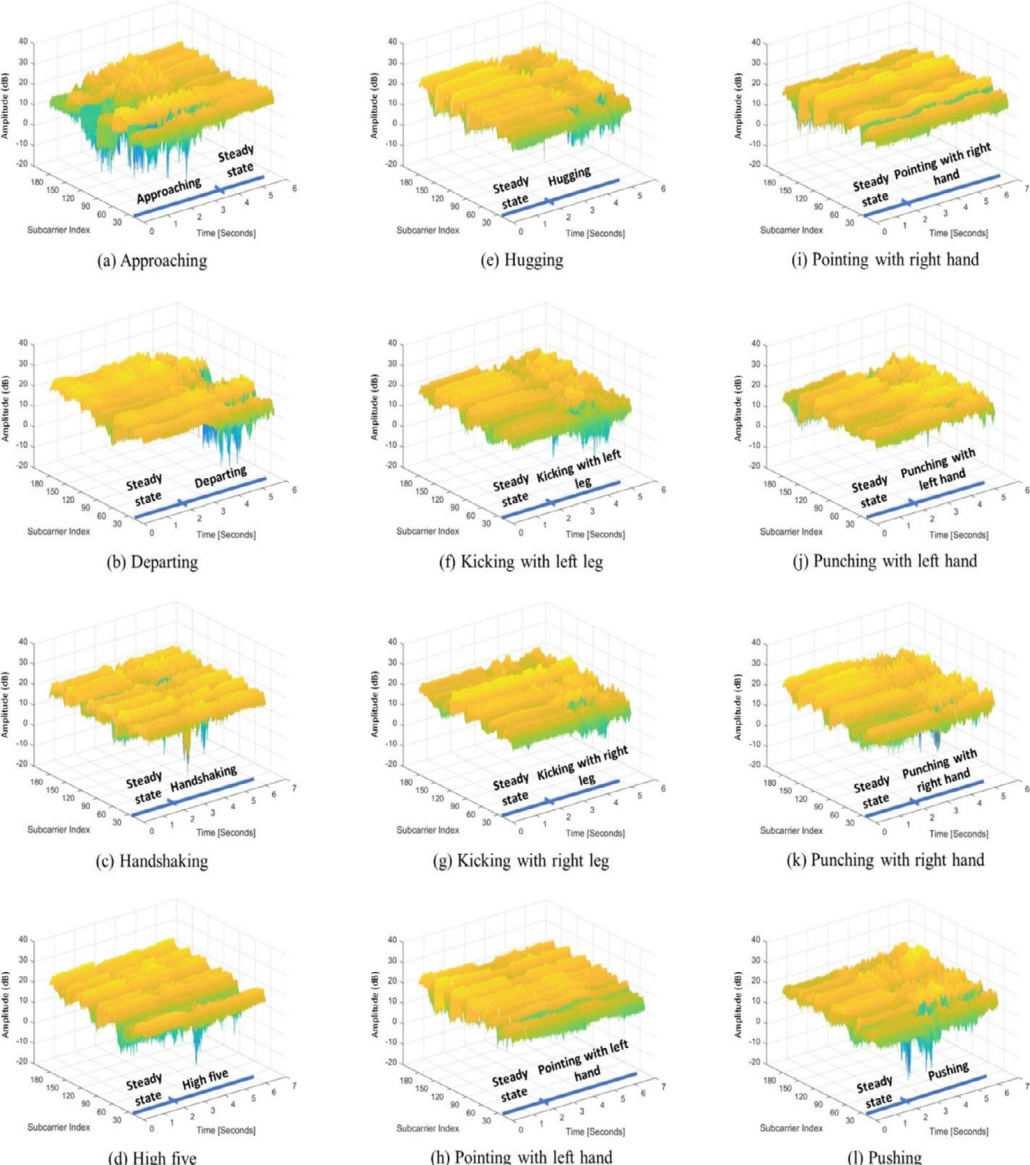
The raw CSI signals of the twelve human-to-human interactions performed by the pair of subjects that constitutes subject 6 and subject 24.

Fig. 2 shows the CSI signals recorded for the pair of subjects that constitutes subject 6 and subject 24 while performing the twelve human-to-human interactions. Moreover, the three-dimensional mesh plots presented in Fig. 2 show the different intervals comprised within each of the twelve interactions, including the steady state and human-to-human interaction intervals.

## 2 Experimental design, materials, and methods

### 2.1 Subjects

A total of 66 healthy subjects (63 males and three females, average ± standard deviation age of 22.1 ± 3.7 years) have volunteered to participate in the experiments. All subjects received a thorough explanation of the experimental procedure. The experimental procedure was conducted according to the Declaration of Helsinki and approved by the research ethics committee at the German Jordanian University. A signed consent form was collected from each subject.

To perform the twelve human-to-human interactions, we have constructed 40 different pairs of subjects from the 66 subjects who have volunteered to participate in this experiment. In particular, the pairs of subjects were constructed according to the following criteria [4]: (1) each subject was selected to be a member of at most two different pairs of subjects, and (2) each subject that was selected as a member of two different pairs has to have different roles in the two pairs, where the role of a subject can be either active role or passive role depending on whether the subject has initiated the interaction or not. Table 2 shows the constructed pairs of subjects along with the role, gender, age, height, and weight of the subjects within each pair.

**Table 2:** The constructed pairs of subjects along with the role, gender, age, height, and weight of the subjects within each pair.

Pair no.	Subjects									
	Active subject					Passive subject				
	Subject ID	Gender	Age (years)	Weight (kg)	Height (cm)	Subject ID	Gender	Age (years)	Weight (kg)	Height (cm)
1	S1	male	20	70	173	S47	male	20	65	175
2	S2	male	18	76	175	S22	male	18	78	172
3	S3	male	22	65	169	S44	male	20	72	174
4	S4	male	21	57	185	S15	male	20	57	185
5	S6	male	20	101	178	S24	male	20	65	179
6	S7	male	23	86	175	S12	male	24	73	184
7	S8	male	21	81	176	S31	male	29	75	171
8	S13	male	20	75	170	S21	male	20	82	176
9	S14	male	26	96	180	S5	male	24	80	165
10	S16	male	23	80	160	S41	male	24	73	175
11	S18	male	27	69	178	S57	male	27	92	180
12	S19	male	18	78	180	S11	male	18	68	168
13	S20	male	29	125	183	S61	male	30	82	179
14	S25	male	22	70	170	S9	male	21	68	167
15	S26	male	18	57	171	S60	male	18	64	168
16	S27	male	20	67	170	S40	male	20	71	174
17	S28	male	28	83	170	S43	male	26	80	174
18	S32	male	27	82	186	S64	male	33	87	190
19	S33	male	22	81	185	S3	male	22	65	169
20	S34	female	21	70	168	S30	female	20	80	160
21	S35	male	20	94	178	S52	male	19	62	170
22	S36	male	26	91	167	S16	male	23	80	160
23	S37	male	19	57	177	S54	female	20	65	171
24	S38	male	19	118	186	S35	male	20	94	178
25	S41	male	24	73	175	S36	male	26	91	167
26	S42	male	23	56	177	S14	male	26	96	180
27	S44	male	20	72	174	S33	male	22	81	185
28	S46	male	29	92	184	S28	male	28	83	170
29	S48	male	20	72	173	S45	male	21	181	73
30	S49	male	18	74	179	S10	male	18	176	86
31	S50	male	21	70	173	S17	male	22	68	172
32	S51	male	20	73	170	S23	male	26	71	175
33	S52	male	19	62	170	S62	male	19	72	178
34	S53	male	34	84	176	S12	male	24	73	184
35	S55	male	21	64	173	S66	male	20	76	180
36	S56	male	22	110	179	S63	male	20	86	178
37	S58	male	21	58	167	S39	male	21	62	174
38	S59	male	20	104	179	S29	male	22	105	179
39	S62	male	19	72	178	S38	male	19	118	186
40	S65	male	19	64	170	S42	male	23	56	177

### 2.2 Experimental procedure

**Fig. 3:**
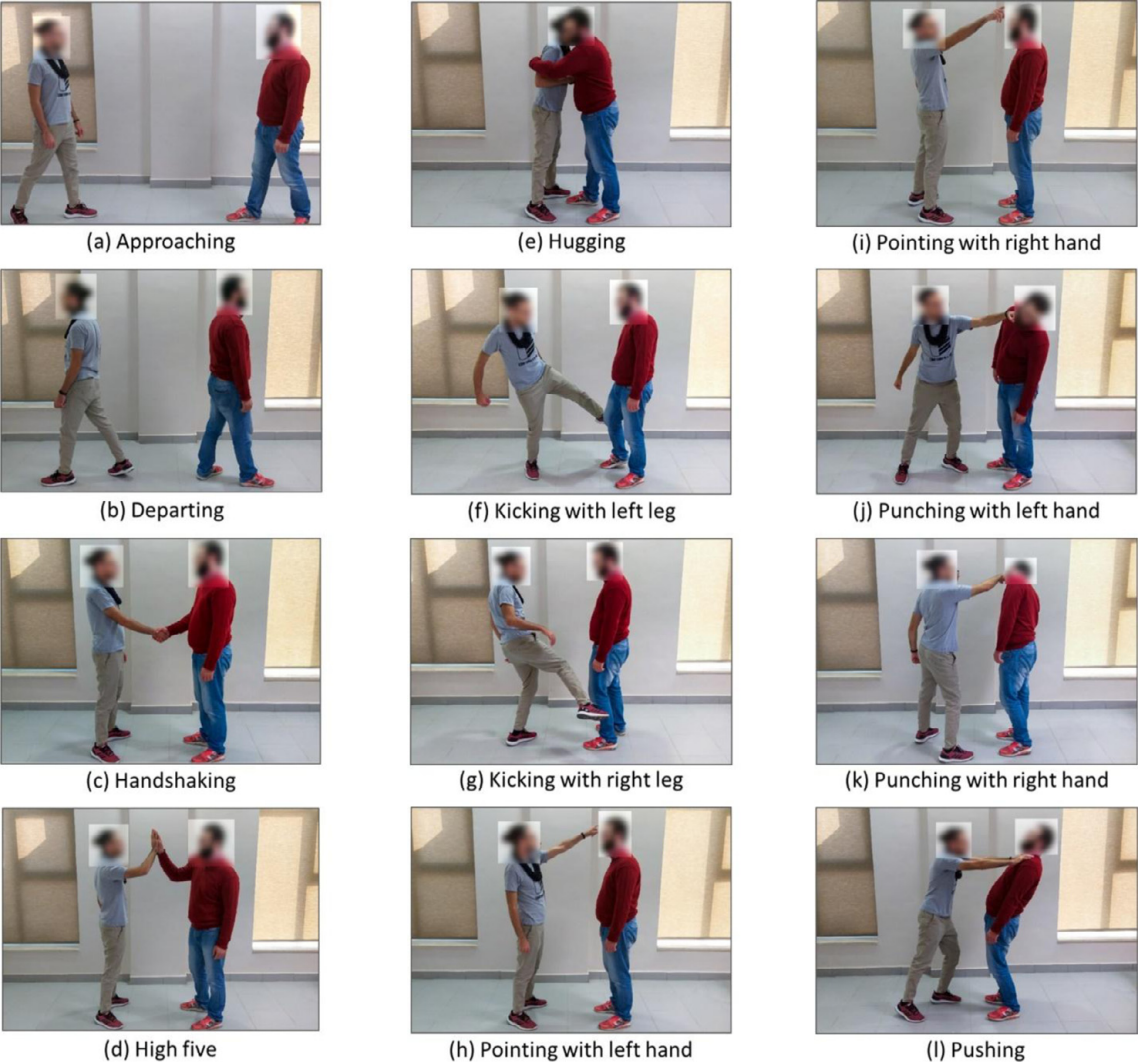
Sample images of the twelve human-to-human interactions considered in our dataset.

Each pair of subjects was asked to perform ten different trials of each of the twelve human-to-human interactions. Fig. 3 shows sample images of the twelve human-to-human interactions considered our dataset. Each of the twelve human-to-human interactions consists of two types of intervals, namely the steady state and the interaction intervals. During the steady state interval, the pair of subjects are standing against each other without performing any activity. On the other hand, during the interaction interval, the pair of subjects perform one of the twelve different human-to-human interactions.

In order to accurately perform the different human-to-human interactions, we have designed twelve timing diagrams that describe how to perform each of the twelve human-to-human interaction. Moreover, we developed a group of pre-programmed beep sounds, where each one of these sounds can be played at a preset time instance to notify the subject to perform a specific action during the time interval following the beep sound. In particular, a short beep sound is used to initiate the recording of each trial, a medium beep sound is used to indicate an interval transition, and a long beep sound is used to announce the end of the recording of each trial. Table 3 shows the timing diagram associated with each of the twelve human-to-human interactions along with the steady state interval, interaction interval, and the time instances associated with the added beep sounds. These timing diagrams were thoroughly explained to the subjects before the beginning of data recording. Moreover, the subjects were asked to follow the timing diagrams during the performance of the twelve interactions and to perform their roles within the amount of time allocated to each interval of a particular interaction.

**Table 3:** The timing diagram associated with each of the twelve human-to-human interactions along with the steady state interval, interaction interval, and the time instances of the added beep sounds. A sound icon is used to mark the locations of the added beep sounds.

Interaction	Timing Diagram
Approaching	
Departing	
Handshaking	
High five	
Hugging	
Kicking with the left leg	
Kicking with the right leg	
Pointing with the left hand	
Pointing with the right hand	
Punching with the left hand	
Punching with the right hand	
Pushing	

### 2.3 Software and equipment

**Fig. 4:**
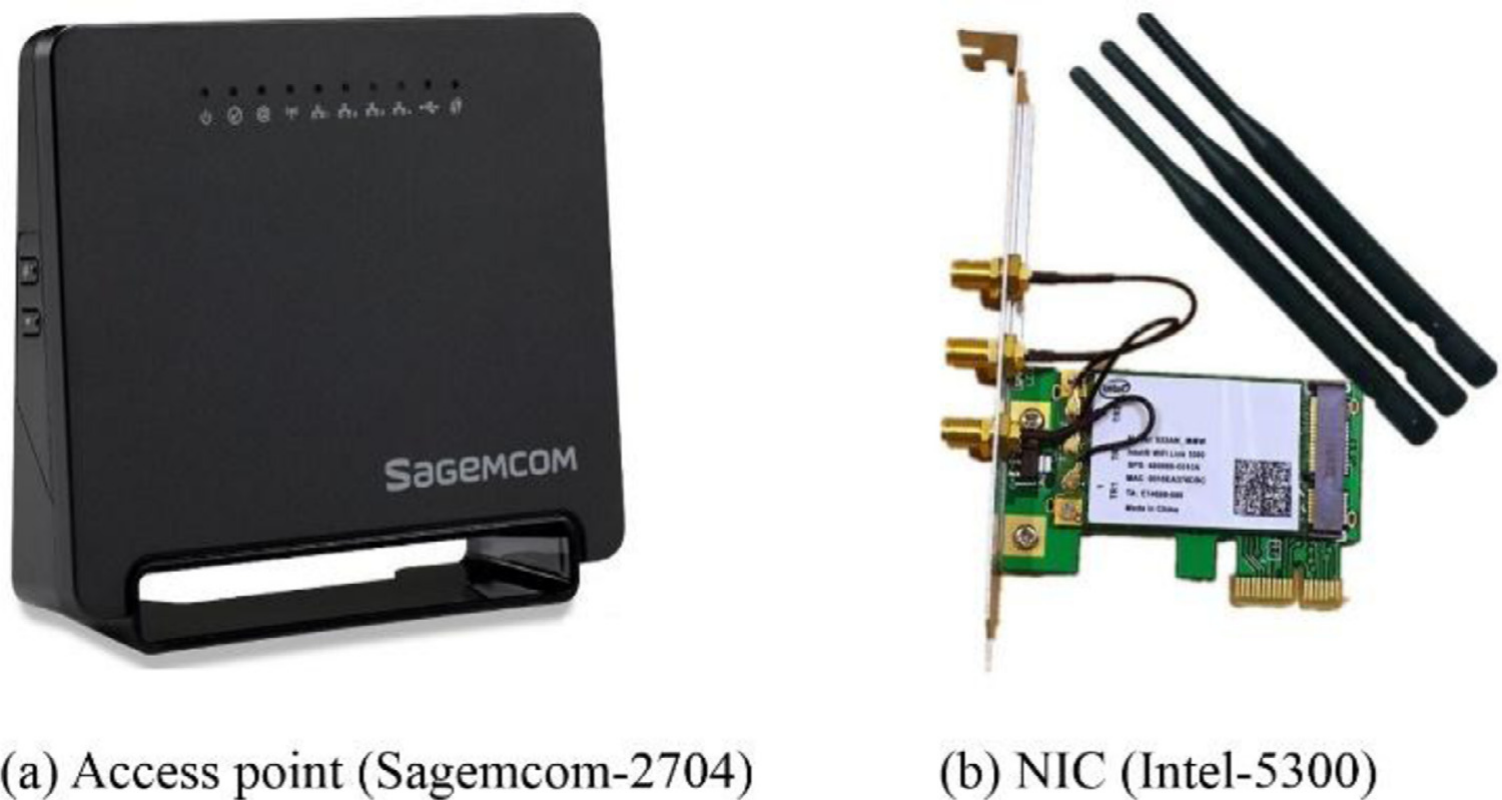
The equipment used for transmitting and receiving Wi-Fi packets.

The publicly available CSI tool [1] was used to record the Wi-Fi signals transmitted from a commercial off-the-shelf access point, namely the Sagemcom 2704 access point, to a desktop computer that is equipped with an Intel 5300 NIC. Fig. 4 shows the utilized access point and NIC. The access point was configured to operate at a frequency band of 2.4 GHz, wireless channel number 6, channel bandwidth of 20 MHz, and modulation coding scheme of index 8. Moreover, the utilized access point and the NIC are compliant with the IEEE 802.11n standard, which supports Multiple Input Multiple Output (MIMO) with the OFDM modulation scheme that allows sending and receiving information over multiple antennas [2,3,5].

**Fig. 5:**
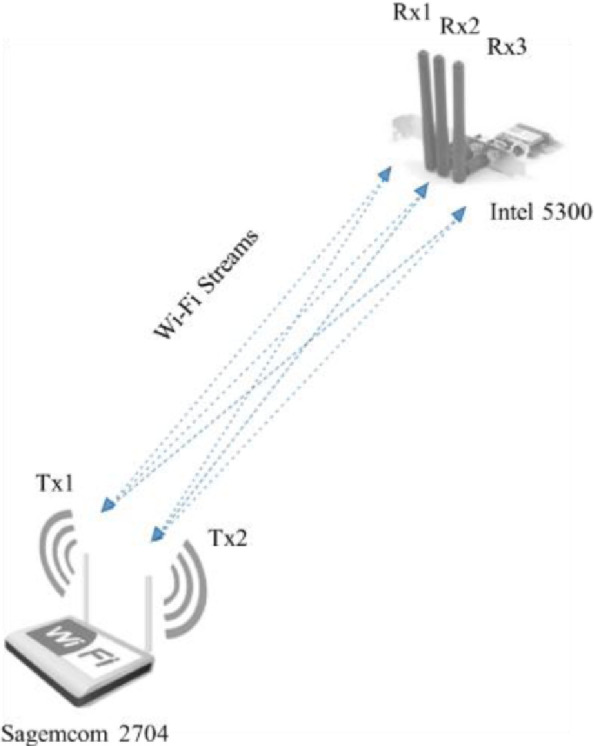
The MIMO streams established between the access point and the NIC. Tx1 and Tx2 represent the two transmit antennas at the access point, while Rx1, Rx2, and Rx3 represent the three receive antennas at the NIC.

The access point comprises two internal transmit antennas (i.e., Ntx=2), and the NIC has three external receive antennas (i.e., Nrx=3). Therefore, the resultant MIMO system consists of 2 × 3 Wi-Fi streams, where each MIMO stream is established between a unique pair of transmit-receive antennas. Moreover, for each OFDM-modulated MIMO stream, the CSI tool is capable of capturing the CSI for 30 subcarriers (i.e., Nsc=30) that are evenly spread over the selected channel bandwidth, which is equal to 20 MHz. Thus, our MIMO system is capable of capturing 6 × 30 subcarriers. Fig. 5 shows the MIMO streams established between the utilized access point and the NIC, which are used to record the CSI while the subjects are performing the twelve human-to-human interactions.

### 2.4 Environment

**Fig. 6:**
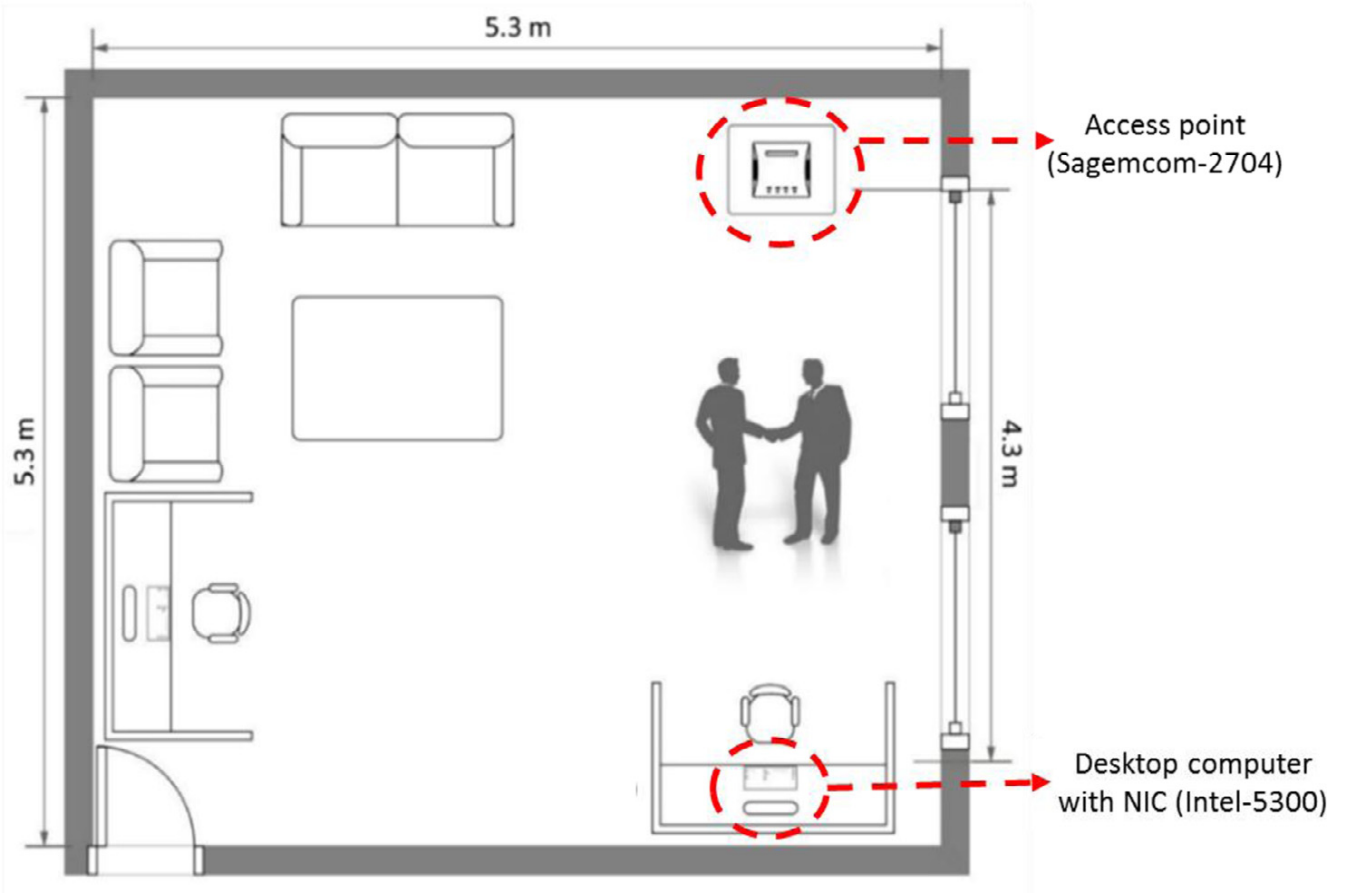
The layout of the room used to collect the data.

The Wi-Fi signals were captured in a furnished room of dimensions 5.3 m × 5.3 m, as shown in Fig. 6. The access point and the NIC were mounted in a line-of-sight configuration at a distance of 4.3 m apart from each other. The pairs of subjects performed the twelve human-to-human interactions in the center of the area located between the access point and the NIC.

## Declaration of Competing Interest

The authors declare that they have no known competing financial interests or personal relationships which have, or could be perceived to have, influenced the work reported in this article.

## Appendix Supplementary materials

Image, application 1
